# Acute myeloid leukemia: does sex matter?

**DOI:** 10.1038/s41375-024-02435-z

**Published:** 2024-10-14

**Authors:** Jesse M. Tettero, Jacqueline Cloos, Lars Bullinger

**Affiliations:** 1 Department of Hematology, Amsterdam UMC, Location Vrije Universiteit, Amsterdam, the Netherlands; 2 Cancer Center Amsterdam, Imaging and Biomarkers, Amsterdam, the Netherlands; 3 Fralin Biomedical Research Institute, Virginia Tech Cancer Research Center, Washington, DC USA; 4 Department of Hematology, Oncology and Cancer Immunology, Charité—Universitätsmedizin Berlin, Corporate Member of Freie Universität Berlin, Humboldt-Universität zu Berlin, Berlin, Germany

**Keywords:** Acute myeloid leukaemia, Cancer genomics

Sex differences are increasingly recognized in the diagnosis and treatment of diseases. Biologically, the primary distinction between females and males is rooted in their sex chromosomes, XX in females and XY in males [1]. While these chromosomal differences account for DNA-based variations, sex-related biological traits are complex and can be influenced by various factors, such as in sex-biased gene-regulatory networks and splicing events that contribute to phenotypic differences between sexes [2, 3]. Epigenetic modifications, such as DNA methylation and histone modification, further influence gene expression differently in females and males, affecting not only physiology but also disease susceptibility [4]. Numerous studies suggest that sex differences also influence outcomes in patients with acute myeloid leukemia (AML). Here, we present the current evidence on sex differences across various aspects of AML.

## Epidemiology

AML is more prevalent in men, with an incidence of around 4.5 per 100,000 males compared to 3.0 per 100,000 females across different age groups [5]. While some studies suggest that males may be diagnosed with AML at a slightly younger age compared to females, the differences are generally small and may not be consistent across all populations [6]. Already in the “premalignant” state of clonal hematopoiesis, sex-specific mutational patterns have been observed in two large lifeline cohorts [7, 8].

## Disease biology

Significant sex differences exist in the genomic aberrations underlying disease pathology, including a higher prevalence of *SF3B1*, *SRSF2*, and *ASXL1* mutations in males and *DNMT3A* mutations in females with clonal hematopoiesis indeterminate potential and myelodysplastic syndromes (MDS) [8]. These differences persist after progression to AML, where female patients more frequently harbor mutations in *DNMT3A*, *FLT3*, *NPM1*, and *TP53*, while mutations in *ASXL1*, *SRSF2*, *U2AF1*, *RUNX1*, and *KIT* are more common in males [8–11]. Additionally, co-mutational patterns differ between sexes as the co-occurrence of *FLT3-*ITD, *NPM1*, and *DNMT3A* mutations was reported overrepresented in females, while males with an *FLT3-*ITD mutation were shown to be characterized by additional mutations in RNA splicing and epigenetic modifier genes [12]. These sex-specific mutational differences may be partly attributed to varying environmental exposures, such as smoking, which has historically been more common in males and is associated with *ASXL1* mutations [13].

In accordance, the impact of sex on the pathophysiology of AML extends beyond genetic mutations. While hormonal influences, particularly estrogen and androgen signaling, may also play an important role in modulating the bone marrow microenvironment and immune response, hormonal effects on leukemogenesis and disease progression are still largely unknown and not well studied yet [14, 15].

## Treatment outcome

The sex differences also translate in prognosis and survival rate differences between female and male AML patients [16, 17]. Although patient outcomes are also influenced by multiple factors such as age, genetic mutations, and comorbid conditions, sex is frequently identified as an independent risk factor. This raises the question of whether sex differences should be integrated into clinical decision-making for AML.

For MDS, the retrospective analysis of the GeoMed consortium including over 13,000 patients demonstrated that outcome prediction scores could be optimized by including sex, thereby resulting in an improved personalized sex-informed approach [18]. Although such a model does not yet exist for AML, it is conceivable that this may also apply. For other cancers, differences in chemotherapy responses and side effect profiles based on sex have been noted [19]. In addition to genomics, hormonal differences (especially during premenopausal phase) and sex-specific pharmacokinetics can contribute to these variations.

In MDS, sex-specific responses to hypomethylating agents might be attributed to underlying biological differences [20]. In AML, sex-stratified analyses from recent key clinical trials reveal diverse responses, as summarized in Table 1. The RATIFY study, a phase 3 trial of the FLT3 inhibitor midostaurin, demonstrated significantly prolonged survival in the cohort of midostaurin-treated patients compared to patients in the placebo arm [21]. However, an analysis stratified for sex (Supplementary Fig. S5A of the original publication) showed a difference in survival with mainly men benefiting from the addition of midostaurin (Hazard Ratio (HR) 0.43, 95% Confidence Interval (CI) 0.28–0.68; *p* < 0.01), while in women there was a trend for the placebo group to perform better (HR 1.42, 95% CI 0.91–2.21; *p* = 0.12). Similarly, the AGILE trial adding ivosidenib to azacitidine in *IDH1*-mutated AML patients found a survival benefit for men [22]. The same trend was found in the VIALE-A trial adding venetoclax to azacitidine, although differences were minor (females: HR 0.68, CI: 0.46–1.02 compared to males: HR 0.62, CI: 0.46–0.85) [23].

**Table 1:** Sex-based outcome differences reported in randomized phase III acute myeloid leukemia (AML) studies.

Study	Study investigation	Endpoint difference	Outcome differences based on sex
Ratify study [21]	Adding midostaurin to standard chemotherapy in *FLT3*-mutant patients	OS	Significantly better OS for midostaurin compared to placebo-treated males (HR 0.43, CI: 0.28–0.68), no difference observed for females (HR 1.42, CI: 0.91–2.21)
AGILE trial [22]	Adding ivosidenib to azacitidine in *IDH1*-mutated patients not fit for intensive therapy	EFS and OS	Significantly better EFS and OS for males treated with ivosidenib compared to placebo (EFS: HR 0.32, CI: 0.13–0.79. OS: HR 0.35, CI: 0.18–0.67), no difference observed for females (EFS: HR 0.35, CI: 0.13–1.00. OS: HR 0.63, CI: 0.31–1.29)
VIALE-A [23]	Adding venetoclax to azacitidine in patients not fit for intensive therapy	OS	Significant better OS for males treated with venetoclax/azacitidine compared to placebo (HR 0.62, CI: 0.46–0.85), no difference observed for females (HR 0.68, CI: 0.46–1.02)
Quantum-First study [24]	Adding quizartinib to standard chemotherapy in *FLT3-*ITD mutated patients	OS	Subgroup analyses demonstrated a significant OS benefit in females (HR 0.69, CI: 0.50–0.96) but not in males (HR 0.86, CI: 0.62–1.19)
ADMIRAL trial [25]	Comparing gilteritinib versus chemotherapy in relapsed or refractory (R/R) *FLT3*-mutated patients	OS	Subgroup analyses demonstrated a significant OS benefit in females (HR: 0.57, CI: 0.40–0.82) but not in males (HR: 0.72, CI: 0.49–1.05)

*CI* 95% confidence interval, *EFS* event-free survival, *HR* hazard ratio, *OS* overall survival, *EFS* event-free survival.

Conversely, there are also studies indicating a survival benefit for female AML patients compared to male patients. For example, the Quantum-First study (testing quizartinib plus chemotherapy for *FLT3-*ITD positive newly diagnosed AML patients) demonstrated benefits mainly in females but not in males [24]. In addition, the ADMIRAL trial adding a more *FLT3*-ITD specific inhibitor gilteritinib to chemotherapy for relapsed or refractory (R/R) *FLT3*-mutated AML showed an overall survival for females, but not for males [25].

The complexity of these findings underscores the necessity for larger studies in AML, based on which a sex-nuanced approach to treating AML can be invented. Personalized medicine, which tailors’ treatment based on individual patient characteristics, should then include not only genetic and molecular profiles, but should also consider sex as a vital factor. A respective precision oncology approach integrating sex-specific differences into therapeutic strategies has the potential to revolutionize AML treatment, thereby ensuring that both female and male patients receive the most effective and appropriate care.

Emerging technologies, such as single-cell sequencing and whole-genome sequencing, offer unprecedented opportunities to delve deeper into the molecular underpinnings of sex differences in AML in the future. By analyzing the tumor microenvironment and the immune landscape at a single-cell resolution, researchers will be able to better uncover sex-specific and potentially hormonally driven cellular interactions and signaling pathways that might drive disease progression and treatment resistance.

In an ideal scenario, future research should focus on designing clinical trials that specifically address sex differences in AML. These studies should not only stratify patients by sex but also be sufficiently powered to investigate the underlying mechanisms driving disparate outcomes, which can be achieved by increased sample sizes or larger collaborative efforts. Meanwhile, preclinical and animal models will be essential for advancing our understanding of how sex hormones and genetic factors influence AML biology and therapeutic responses.

In conclusion, disease biology and AML outcomes differ significantly between female and male patients. This highlights the importance of considering sex as a factor in AML management, especially with the recent increase in novel targeted strategies. Ultimately, prognostication and therapeutic strategies in AML could be improved by incorporating sex-specific considerations. Thus, we urge researchers to include sex-stratified analyses in their current studies to uncover the extent and relevance of differences, and to enable a more solid data basis.
